# Cocoa

**Published:** 1885-04

**Authors:** 


### COCOA.

The *Journal of Inebriety* says: After the removal of the alcohol in the treatment of the inebriate, a stage of profound exhaustion and neurasthenia comes on. An unmasking, as it were, of a nameless variety of neuralgias and states of irritation, both physical and psycical, which tax therapeutic resort to its utmost to meet. Functional changes and perversions that are intense, complex and very changeable, associated with organic lesions, both obscure and well defined, difficult to diagnose, and more difficult to cure. These are termed, in general, states of brain and nerve exhaustion and the usual remedies are quinine, strychnine, electricity, baths, nutrients and other general remedies. The materia medica is constantly searched for tonics that will lessen this neurasthenic stage, and enable the patient to regain in some measure his lost control of mind and body, and rise above the mental depressions so common and agonizing. Our exprerience with cocoa in many cases seems to indicate that it meets these wants more positively than any other now used. Through the kindness of Parke, Davis & Co., of Detroit, we were supplied with the fluid extract of cocoa leaves, a preparation of known purity and value. The results of its use in these cases may be summed up as follows: 1. After the removal of alcohol, cocoa in doses of from one-half to one ounce every four hours was speedily followed by the most characteristic symptoms of improvement. 2. Its action on the brain and nervous system was that of an exhilarant and slight narcotic, relieving depressions and lessening irritable nerve conditions. In cases of organic and functional lesions of the heart, an increased steadiness of pulse-beat and diminution of pulse irritability was apparent. 3. The psychological depressions and neuralgias so common in this stage were lessened, and disappeared altogether in most cases, especially for some time after the use of the drug. 4. Both the appetite and sleep, in all cases where it was given freely, improved rapidly, and the future of these cases seemed to have less complications and more positive recoveries. 5. No evidence was brought out that would indicate it destroyed the cravings for alcohol in cases of dipsomania; but it was clear that given freely in these cases it lessened the intensity and duration of the attack. The conclusion which was indicated by this study was that cocoa was a tonic of great value in inebriety, and more nearly a specific than other remedy now known.

Dr. W. Coles (*Weekly Medical Review*) says he has prescribed cocoa quite frequenily within the last few years, and in many instances with most gratifying results. Its property of retarding thirst, while at the same time acting as a cerebral stimulant, renders it a most excellent substitute for ardent spirits, or beer, especially when persons have been indulging too freely and are attempting to break off the habit. It seems to strengthen will power by removing that shakiness and mental depression which is one of the incentives with the inebriate to return to his cups. He has been told by intelligent patients that this drug certainly seems to possess the virtue of contributing to the relief of that longing which at times overtakes the drunkard with such overwhelming force. When administered under such circumstances it promotes a sense of composure, which enables the patient to sleep, whereas, when in health, the same dose would aid his faculties in keeping awake.

